# Towards ready-to-use 3-D scaffolds for regenerative medicine: adhesion-based cryopreservation of human mesenchymal stem cells attached and spread within alginate–gelatin cryogel scaffolds

**DOI:** 10.1007/s10856-013-5108-x

**Published:** 2013-12-03

**Authors:** Alisa Katsen-Globa, Ina Meiser, Yuriy A. Petrenko, Roman V. Ivanov, Vladimir I. Lozinsky, Heiko Zimmermann, Alexander Yu. Petrenko

**Affiliations:** 1 Department for Biophysics and Cryotechnology, Fraunhofer Institute for Biomedical Engineering, Ensheimer Str. 48, 66386 St. Ingbert, Germany; 2 Institute for Problems of Cryobiology and Cryomedicine NAS Ukraine, 23 PeryaslavskayaStr, Kharkiv, 61015 Ukraine; 3 A.N. Nesmeyanov Institute of Organoelement Compounds, Russian Academy of Sciences, Vavilov Street, 28, 119991 Moscow, Russian Federation; 4 Chair of Molecular and Cellular Biotechnology/Nanotechnology, Saarland University, PO Box 151150, 66041 Saarbrücken, Germany

## Abstract

Cultivation and proliferation of stem cells in three-dimensional (3-D) scaffolds is a promising strategy for regenerative medicine. Mesenchymal stem cells with their potential to differentiate in various cell types, cryopreserved adhesion-based in fabricated scaffolds of biocompatible materials can serve as ready-to-use transplantation units for tissue repair, where pores allow a direct contact of graft cells and recipient tissue without further preparation. A successful cryopreservation of adherent cells depends on attachment and spreading processes that start directly after cell seeding. Here, we analyzed different cultivation times (0.5, 2, 24 h) prior to adhesion-based cryopreservation of human mesenchymal stem cells within alginate–gelatin cryogel scaffolds and its influence on cell viability, recovery and functionality at recovery times (0, 24, 48 h) in comparison to non-frozen control. Analysis with confocal laser scanning microscopy and scanning electron microscopy indicated that 2 h cultivation time enhanced cryopreservation success: cell number, visual cell contacts, membrane integrity, motility, as well as spreading were comparable to control. In contrast, cell number by short cultivation time (0.5 h) reduced dramatically after thawing and expanded cultivation time (24 h) decreased cell viability. Our results provide necessary information to enhance the production and to store ready-to-use transplantation units for application in bone, cartilage or skin regenerative therapy.

## Introduction

3-D substrates have great impact in the development of scaffolds for tissue engineering in regenerative medicine [1–6], especially in combination with multipotent stem cells, like human mesenchymal stem cells (hMSCs), that are capable to differentiate in various cell types of mesoderm germ layer [7]. These scaffolds provide native environments required for cell proliferation and differentiation at an optimal growth-area-to-volume-ratio. Among the numerous kinds of materials serving as scaffolds, alginate convinces by following advantages for medical applications: the natural polysaccharide forms a hydrogel when crosslinked with multivalent cations, is cyto- and biocompatible, as well as biodegradable [8–10]. Furthermore, pore sizes in alginate scaffolds can be adjusted and allows immuno-isolation of encapsulated grafts along with nutrient supply and metabolic factor diffusion [11–15] as well as cell motility and contact in scaffolds with large, open pores [16–19]. Latter are often fabricated by cryogenic methods, like freeze-drying and cryostructuring, that produced a macro-porous cryogel scaffolds by large ice crystals at low cooling rates and temperature up to −20 °C [16, 18–24].

However, untreated alginate is not famous for cell adhesion, especially hMSCs remain round-shaped with little attachment sites caused by missing integrin-based cell-substrate binding [25]. To enable or enhance cell adhesion, alginate scaffolds are coupled with different extracellular matrix (ECM) proteins such as collagen/gelatin, fibronectin, laminin [26]. Recently we proposed a chemical binding of gelatin to walls of alginate sponges, promoting hMSCs adhesion, growth and proliferation without interrupting of adipogenic, chondrogenic and osteogenic differentiation [19]. The bio-, and cytocompatibility of these alginate-gelatin scaffolds, as well as other alginate-cryogel compositions, have already been shown: they exhibit good adhesion, growth, and proliferation characteristics for various cell types and have a high potential to serve as matrix for tissue engineered transplantation units [23, 27–31].

Application of cell-scaffold constructs in regenerative medicine implies a linear workflow from cell seeding in appropriate scaffolds over proliferation in vitro to transplantation in vivo, that does not allow pausing or even storing the tissue constructs in biobanks for future supply. Cryopreservation of hMSC-scaffold constructs with maintained cell viability and functionality is a desirable approach [32–34] to overcome shortage in supply and would allow immediate application of the constructs by their ready-to-use character. Even differentiation in various tissue types in situ is possible [32, 33, 35, 36]. In spite of several decades of research, it is still very difficult to cryopreserve adherent cells. The cells with cell–cell and cell-substrate contacts are much more sensitive to freeze–thaw injury than single cells in suspension, their spacious plasma membrane and cytoskeleton is affected by mechanical ruptures, followed by cell detachment and death [36–40]. These contacts mediated by cytoskeleton proteins, are involved in anchorage, spreading and motility of adherent cells [41], however, it wasn’t still shown an influence of cell spreading on the cryopreservation success. Attachment and spreading processes depend on the duration of cultivation and can already be detected after a few hours [42]. To enhance cryopreservation success, the strained cytoskeleton of adherent cells has to be protected against injury caused by freezing and thawing procedures. Since it is well known, that water molecules are the main cause of cryoinjury (solution effects, mechanical damage by ice crystals) [43], hydrogel scaffolds like alginate seem to have beneficial effects for cryopreservation procedures. Here, we show that alginate-gelatin scaffolds are suitable for 3-D cultivation and cryopreservation of hMSCs and can serve as ready-to-use scaffold constructs for regenerative medicine. It was for the first time demonstrated that cell recovery after thawing depends on cultivation time before cryopreservation: short cultivation time had a beneficial effect. This was analyzed with confocal scanning electron microscopy (CLSM) for viability and cell number. Additionally, cell functionality by spreading and motility on the scaffolds as well as cell injury caused by cryopreservation was analyzed by scanning electron microscopy (SEM), where surface changes and cell spreading on the non-transparent substrate can be monitored and quantified [44, 45].

## Materials and methods

### Preparation of scaffolds

**Fig. 1:**
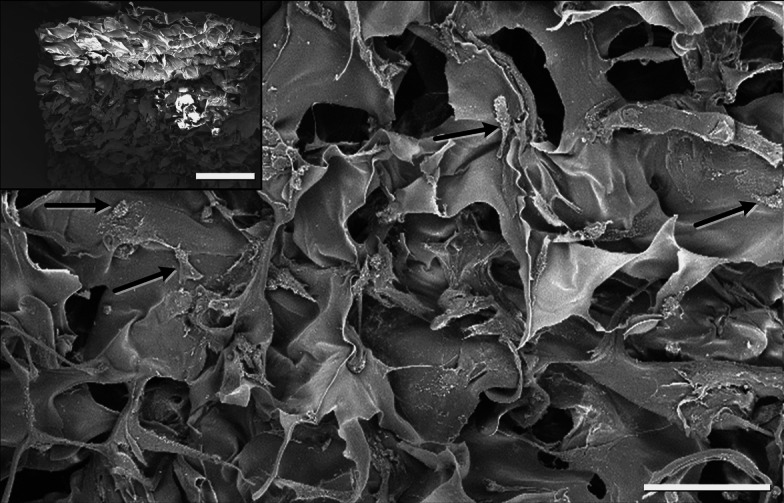
Representative SEM-image of an alginate-gelatin cryogel scaffold with adherent hMSCs (indicated by *black arrows*). *Scale bar* 100 μm. *Inset* (*top*, *left*) shows an overview. *Scale bar* 500 μm

The wide pore alginate-based scaffold matrix (Fig. 1) was prepared essentially in accordance with the patented method described in [46]. In brief, sodium alginate of trade mark Manugel DMD (ISP, USA) was dissolved in distilled water in the concentration of 1.5 wt%. The solution was poured in plastic Petri dishes (40 mm in dia.; the layer thickness was 2 mm) and placed on to the strictly horizontal platform located in the chamber of precision programmable cryostat FP 45 MH (Julabo, Germany). The samples were frozen at −15 °C for 3 h, further they were transferred into the freeze-drier Alpha 1–2 LD plus (Martin Christ, Germany) and lyophilized for 24 h. Dry wide-porous Na-alginate disks were obtained, which were then immersed in saturated ethanolic solution of calcium chloride (Panreac Quimica SA, Spain) and kept there for 3 days with periodical, gentle shaking in order to exchange Na^+^-ions for the cross-linking Ca^2+^-ions. Further, the wide-porous Ca-alginate disks were rinsed with medical ethanol several times for salt removal. Subsequently, scaffold modification was carried out according to the procedure reported elsewhere [19]. Briefly, the disks were soaked in 0.1 M aqueous solutions of calcium hydroxide (Panreac Quimica SA, Spain) for 30 min with simultaneous argon bubbling. Afterwards, divinylsulfon (DVS) (Sigma-Aldrich Chemie GmbH, Germany) was added to 0.75 wt% DVS concentration in the liquid phase. Further the disks were incubated in DVS-medium for 18 h at room temperature with periodical shaking for grafting reactive vinyl-sulfone groups to the polysaccharide core. Then the disks were rinsed with water from the unreacted DVS residues, soaked in 1 wt% aqueous solution of gelatin (gelatin from porcine skin, type A (G_A_), Sigma, USA) and adjusted to pH 9 with calcium hydroxide. Now, the disks were incubated in this medium for 12 h at 40 °C with gentle stirring followed by rinsing with warm (40 °C) water till the absence of protein in the washing wastes. Finally, water was substituted for ethanol, under which the resulting scaffolds were stored (4–6 °C) before use.

### Cell cultivation

Umbilical cord hMSCs were obtained from Promocell (Heidelberg, Germany) and cultivated according to manufacturer’s protocol. Before seeding, the scaffolds were cut manually to a size of ~5 × 5 × 2 mm and placed into culture medium for 15 min. After culture medium removal, the scaffolds were transferred into non-adhesive Petri dishes. To each scaffold, 100 μL with 10^5^ cells in total was added manually on top. The following time periods of cell cultivation were chosen for non-frozen control and cryopreserved samples: 0.5, 2 and 24 h. After 0.5 h cultivation the sponges in group 0.5 h were stained and analyzed, and culture medium was added to other groups. For each time point three scaffolds were used and each experiment was repeated three times (*n* = 3). To estimate cell shrinkage during cell preparation for SEM, cells were also seeded on 35 mm ibidi plastic μ-dish with grid (ibidi GmbH, Martinsried, Germany) so exactly the same cells could be analyzed using different methods.

### Cryopreservation and recovery

For cryopreservation each scaffold was placed into one cryovial (Sigma-Aldrich, Taufkirchen, Germany) equipped with cryo-medium contains 10 % dimethyl sulfoxide (DMSO) in culture medium. After 5 min incubation at 4 °C, the vials were transferred into Mr. Frosty-Boxes (Nalgene, Sigma-Aldrich, Taufkirchen, Germany) to guarantee a cooling rate of 1 °C/min and into a −80 °C freezer overnight. Next day the samples were transferred into the vapor phase of liquid nitrogen (<−160 °C) and kept there for at least 24 h. The samples were thawed in a 37 °C water bath, and the scaffolds were placed into non-adherent well-plates containing 0.5 ml of Dulbecco’s phosphate buffered saline (PBS) with Ca^2+^ and Mg^+^ (Invitrogen, Karlsruhe, Germany) by forceps. The PBS was changed once, removed and substituted with culture medium. Immediately after thawing some samples were stained for viability, the others were placed into incubator for recovery. Recovery time points were 0, 24 and 48 h after thawing. For each time point three scaffolds were used and each experiment was repeated three times (*n* = 3).

### Cell membrane integrity and metabolic activity assay

The membrane integrity and metabolic activity was assessed using a double fluorescent staining with enzyme substrate fluorescein diacetate (FDA) and DNA-dye ethidium bromide (EB) as previously described [47]. FDA is non-fluorescent but after crossing the plasma membrane of metabolic active cells by diffusion it is hydrolyzed to fluorescein, indicating viable cells. EB can only enter cells with damaged membranes, intercalates with their DNA and exhibit a red fluorescence. FDA/EB staining was analyzed by CLSM (Eclipse EZ-C1, Nikon GmbH, Düsseldorf, Germany). Confocal images were obtained along the Z-axis with 20 μm intervals at excitation wavelength of 488 nm (FDA) and 543 nm (EB).

### SEM

To evaluate cell morphology and behavior within the scaffolds, SEM was performed before and after freezing and recovery. Adherent hMSCs on the alginate-gelatin scaffolds were prepared as previously described [48] with some modification for alginate preparation. Briefly, the samples were fixed in 2 % glutaraldehyde in sodium cacodylate buffer, treated with 2 % osmium tetroxide and 1 % tannic acid in 0.15 M sodium cacodylate buffer. After dehydration in increasing alcohol concentration, the samples were dried in hexamethyldisilazane (HMDS, Sigma-Aldrich, Taufkirchen, Germany). Finally, the samples were coated with carbon, ion-sputtered with gold–palladium and studied in field emission scanning electron microscope Phillips FESEM XL30 (FEI, Eindhofen, Netherlands) by 5 and 10 kV accelerating voltage and 10 mm working distance.

### Image analysis

#### Determination of cell viability

**Fig. 2:**
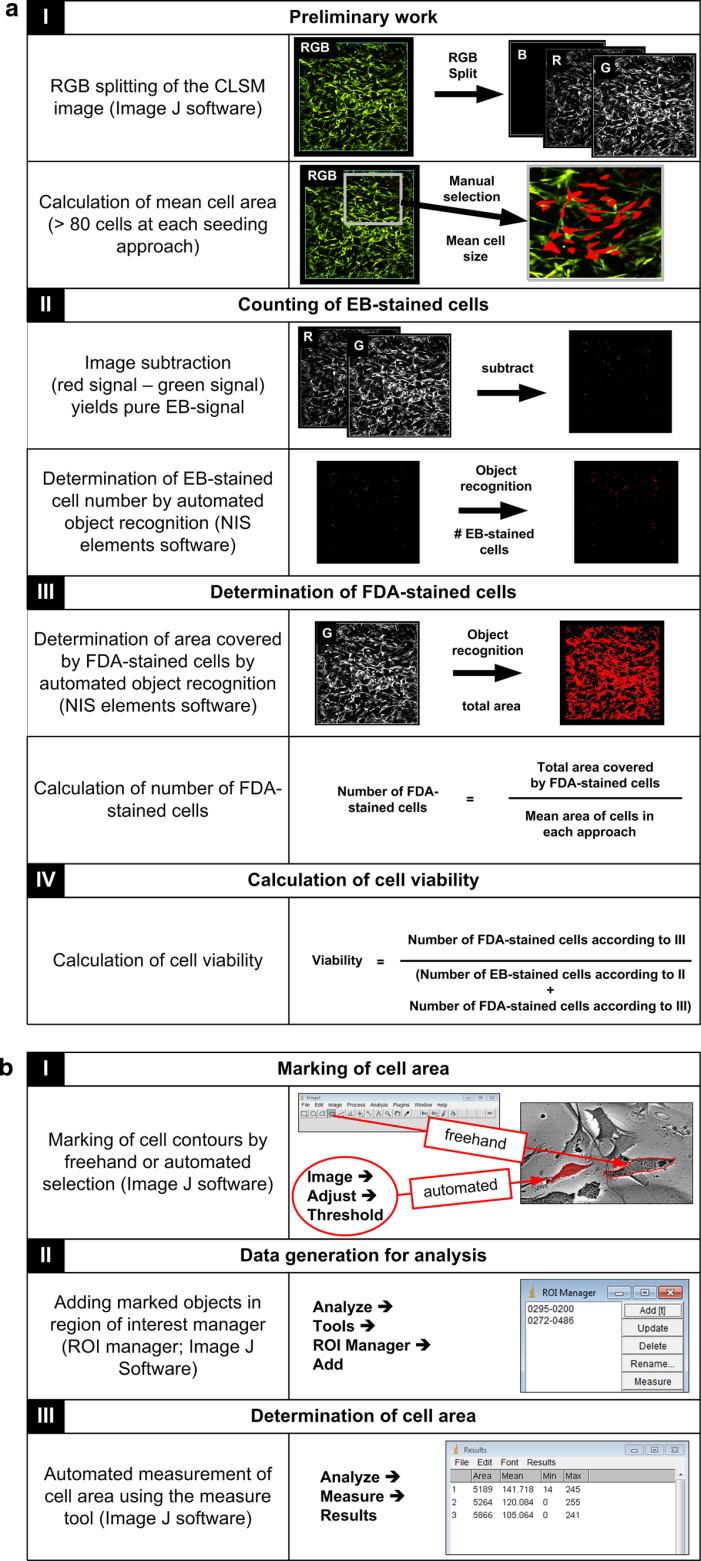
Image analysis steps: **a** Image analysis for viability estimation after cell membrane integrity and metabolic activity assay with Image J and NIS elements software. **b** Determination of cell area with Image J software

Analysis steps of CLSM fluorescence images for viability estimation are presented in Fig. 2a. Two programs were used: Image J software (NIH, Bethesda, MD, USA) and NIS Elements imaging software (Nikon GmbH, Düsseldorf, Germany). Image J program was used to split CLSM-images in color channels and subtracted them to obtain pure green and red signals. Afterwards the mean single cell size was obtained by measuring the area of at least 80 green-fluorescing cells in NIS elements. The estimation of vital cell number was performed by dividing the total green area of one image, which was also obtained by NIS elements, by the mean single cell size. The number of dead cells per image was obtained from object count in red signal image, since EB-signals are only small dots. Finally, viability was calculated by dividing the number of living cells by the total number of cells. Additionally, we calculated the total number of attached living cells by standardization to total cells in control.

#### Determination of cell spreading area and cell shape

For hMSCs spreading estimation we used previously developed SEM-method based on the determination of cell area in backscattered electron (BSE) mode of SEM [45]. Due to overlapping and sometimes minor differences of brightness/contrast between scaffolds and cells, in this work we measured the cells not only automatically, but also manually by freehand selection and analyzed/measured the cell area in Image J software (see Fig. 2b). More than 90 cells per approach were measured. Total number of cells at each time point was 300–400. From the same SEM-images we estimated the number of round-, or flower-shaped and star-shaped cells, since the work of Kilian et al. [49] shows that the cell shape can reflect their future differentiation possibilities.

#### Determination of cell shrinkage by SEM preparation

After SEM preparation (see above Sect. 2.5) and before dehydration, hMSCs cultivated 0.5, 2 and 24 h on the ibidi-plastic μ-dishes were photographed under water layer. The exact same cells were found and measured after dehydration and HMDS-drying in SEM. The areas of spread cells were determined in light microscopic (LM)-, and SEM-images with above mentioned and described method. A cell shrinkage coefficient of 1.35 ± 0.25 (*n* = 298) was calculated by dividing cell area measured in LM images by the corresponding cell area measured in SEM images. This coefficient was taken into account for our cell area determination.

### Statistical analysis

Results of statistical analysis are presented as mean ± standard deviation, evaluated with paired Student *t* test and were considered significant by *P* < 0.05.

## Results

### Effect of cultivation time on the cell viability and recovery after cryopreservation

**Fig. 3:**
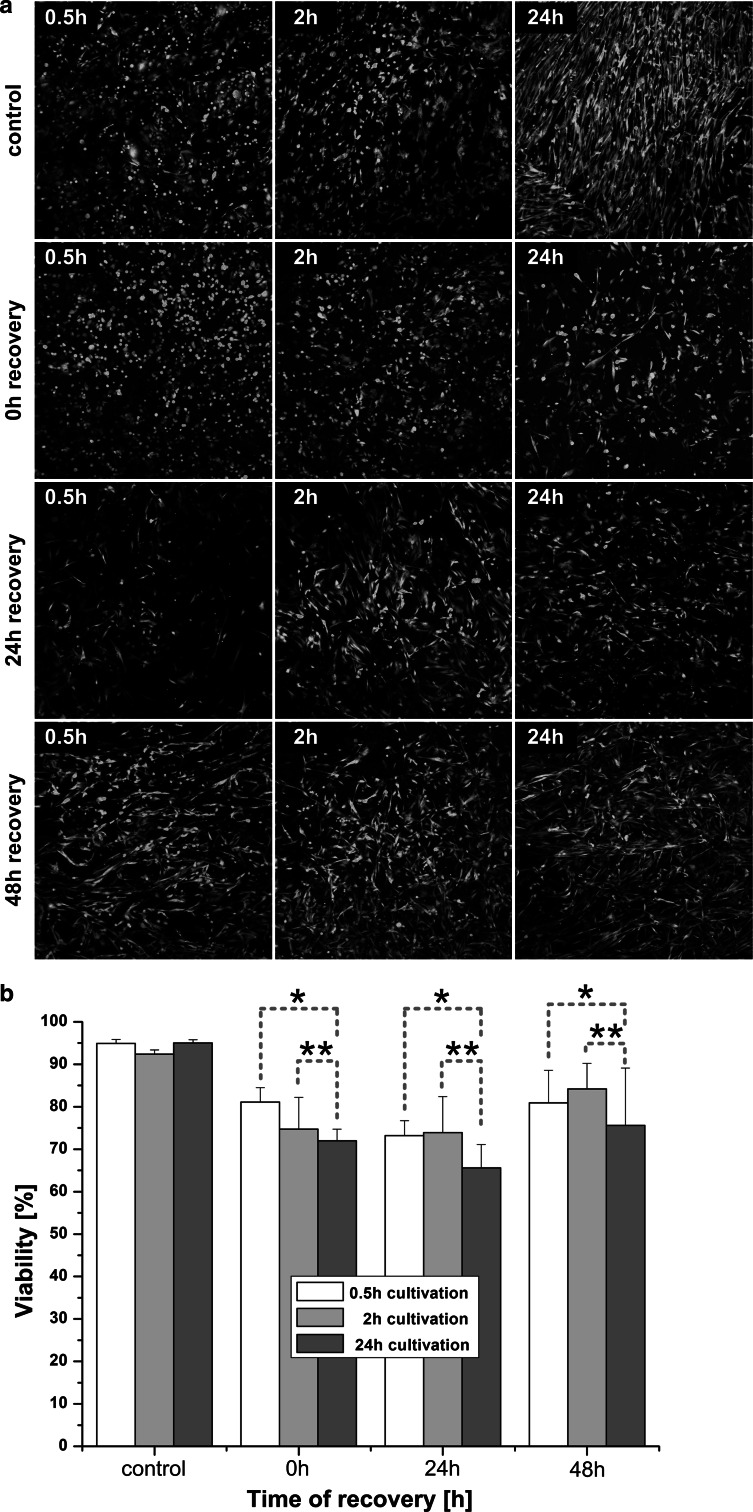
Viability determination: **a** Representative CLSM-images of cell membrane integrity and metabolic activity assay (*n* = 3). **b** Calculated viability of hMSCs cryopreserved adherently within alginate-gelatin scaffolds. The groups 0.5 versus 24 h (*) as well as 2 versus 24 h cultivation (**) indicate statistical significance at all recovery times (*P* < 0.05)

Figure 3a shows representative CLSM fluorescence images of hMSCs before and after freezing-thawing procedure, cultivated for different time periods. The living (green) and damaged (red) cells are well distinguishable. More red cells were seen in scaffolds cultivated for 24 h prior to cryopreservation than in scaffolds cultivated for 0.5 and 2 h, respectively: viability decreased immediately after thawing and remained significant lower as in other groups after 24 and 48 h recovery (see Fig. 3b). In every approach, adherent and living cells were recovered after freezing-thawing procedure (see Table 1), whereas in the 2 h cultivation group cells recovered most rapidly from freezing stress. The numbers of attached living cells in relation to the total cell numbers, displayed in Table 1, also showed best results in samples cultivated for 2 h prior to cryopreservation: hardly any changes immediately after thawing, slight loss of cells after the following 24 h recovery, but afterwards a complete recovery and even an increase in cell number after 48 h. In contrast, cell number in the 0.5 h cultivation group decreased about 50 % after thawing. However, the total cell number of the non-frozen control after 2 h cultivation was significantly less in comparison with 0.5 and 24 h.

**Table 1:** Calculated number of adherent cells (*n* = 3)

Time of recovery (h)	0.5 h		2 h		24 h	
	Mean number living cells	Total cells evaluated	Mean number living cells	Total cells evaluated	Mean number living cells	Total cells evaluated
Time of cultivation (h)^a^						
Control	20,253 ± 377*	21,225 ± 385	10,809 ± 200**	11,630 ± 205	21,227 ± 327*	22,146 ± 332
0 h	13,577 ± 271*	16,385 ± 294	11,608 ± 155**	14,763 ± 164	10,089 ± 187*	13,767 ± 225
24 h	8,738 ± 142	11,805 ± 170	9,121 ± 178	12,023 ± 210	10,340 ± 170	15,693 ± 244
48 h	10,896 ± 185	13,328 ± 199	13,050 ± 162	15,485 ± 179	15,728 ± 261	20,123 ± 232
Mean number of living cells, normalized to control (%)^b^						
0 h	63.97 ± 1.99		99.81 ± 1.34		45.56 ± 1.85	
24 h	41.17 ± 1.63		78.43 ± 1.95		46.69 ± 1.64	
48 h	51.34 ± 1.69		112.21 ± 1.24		71.02 ± 1.66	

^a^Mean number of living and total evaluated cells

^b^Normalized number of living cells to control (%)

* Cell number recovered after 0 h were statistically significant less than cell number adherent on non-frozen control cryogels after 0.5 h and 24 h cultivation (*P* < 0.05)

** Difference in cell number before and after cryopreservation of 2 h cultivation cryogels was statistically not significant (*P* < 0.05)

### Cell surface and cell-substrate behavior before and after cryopreservation procedure

**Fig. 4:**
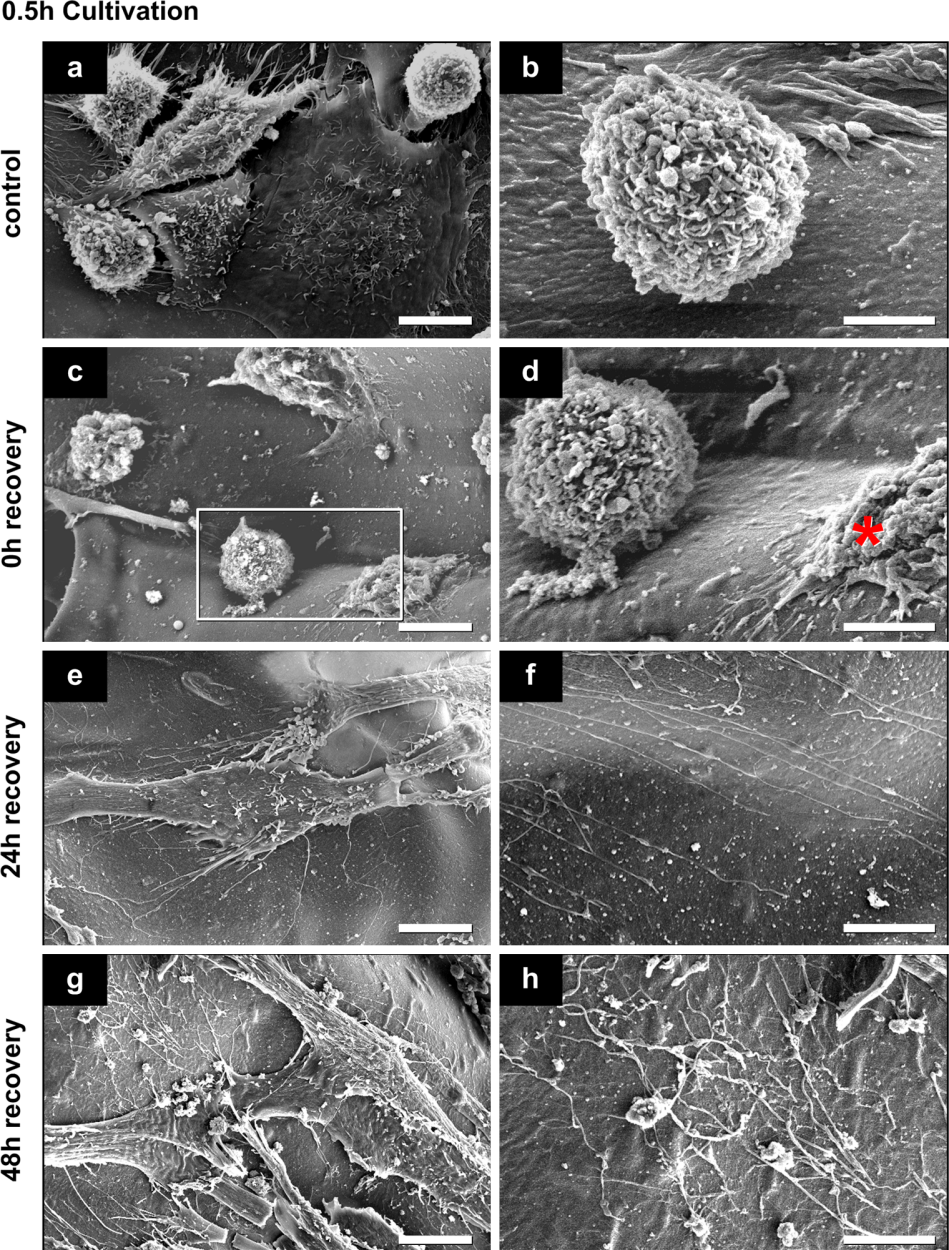
Representative secondary electron SEM-images of hMSCs surface features and behavior before and after cryopreservation after a 0.5 h cultivation (*n* = 3). **d** *Rectangle *marked part of image shown in **c**. Damaged cell in **d** is marked with *red asterisk*. *Scale bars* in **a**, **c**, **e** and **g** indicate 10 μm; in **b**, **d**, **f** and **h** 5 μm (Color figure online)

**Fig. 5:**
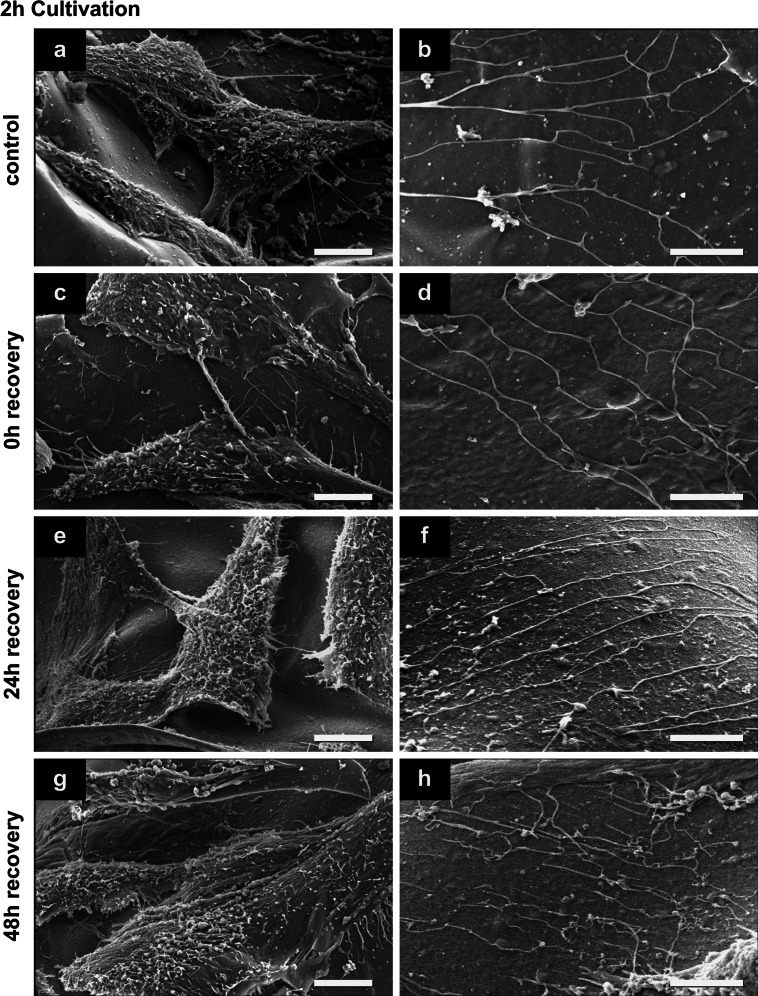
Representative secondary electron SEM-images of hMSCs surface features and behavior before and after cryopreservation after a 2 h cultivation (*n* = 3). *Scale bars* in **a**, **c**, **e** and **g** indicate 10 μm; in **b**, **d**, **f** and **h** 5 μm

**Fig. 6:**
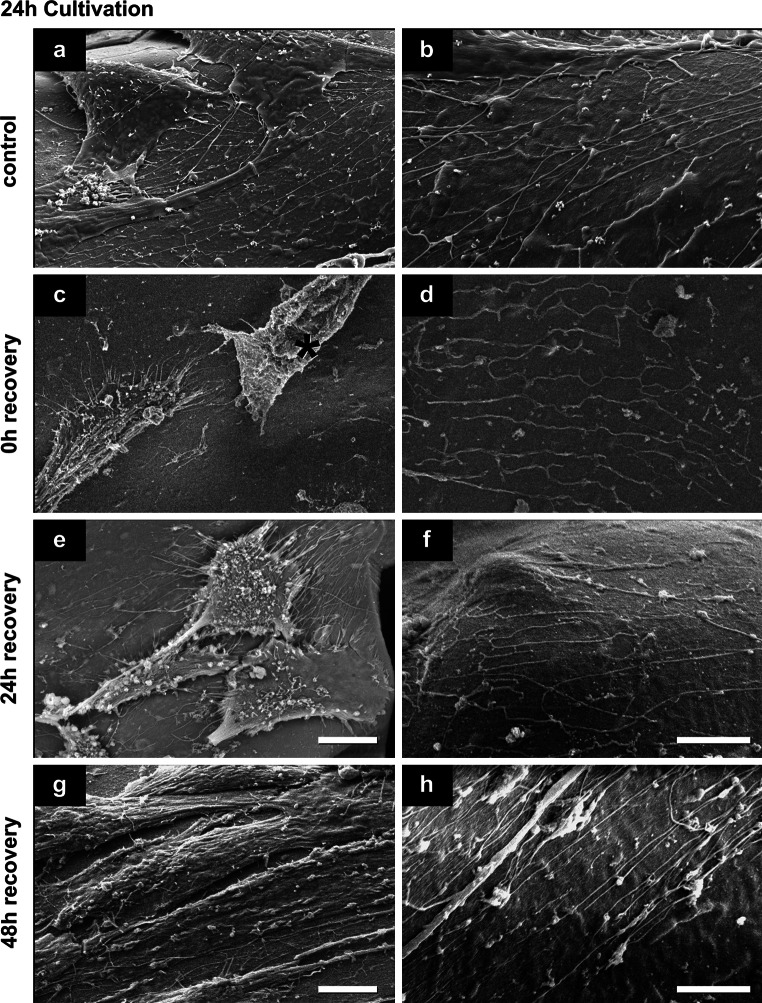
Representative secondary electron SEM-images of hMSCs surface features and behavior before and after cryopreservation after a 24 h cultivation (*n* = 3). Damaged cell in **c** is marked with *red asterisk*. *Scale bars* in **a**, **c**, **e** and **g** indicate 10 μm; in **b**, **d**, **f** and **h** 5 μm (Color figure online)

SEM investigation of hMSCs in non-frozen control and after cryopreservation indicated good attachment and spreading of hMSCs on the alginate-gelatin scaffolds (Figs. 4, 5, 6). The scaffolds were smooth and showed no ruptures or damages after cryopreservation. Spread cells in control were mostly covered with microvilli and some small vesicles (Figs. 4a, 5a, 6a), the round cells have also some small blebs/vesicles and ruffles (Fig. 4b). After 0.5 h cultivation nearly half of the cells in control were round or flower-shaped (Fig. 4a, b). After 2 h cells were more spread (Fig. 5a) and star-shaped, in some cases round and flower-shaped. After 24 h cultivation, hMSCs were well spread, flat, and star-shaped with tight cell–cell and cell-substrate contacts (Fig. 6a). Thin long fibers (up to 200 nm in dia.) were seen in control on the scaffold almost everywhere, except in samples after 0.5 h cultivation (Figs. 5b, 6b).

Immediately after thawing (0 h recovery) of the scaffolds, cultivated for 0.5 h before cryopreservation, round and flower-shaped cells with same surface features like cells in control were observed (compare Fig. 4b, d). The surface of some cells in samples after 0.5 and 24 h cultivation has changed: next to vital cells with surfaces comparable to control hMSCs, damaged spread cells with disruptions or cells with crinkled plasma membranes were seen (cells marked with red asterisk in Figs. 4d, 6c). In samples after 24 h cultivation prior cryopreservation traces of detached cells were sometimes visible (data not shown). Most hMSCs after 2 h cultivation had the similar surface morphology, cell–cell and cell-substrate contacts as in control (compare Fig. 5a, c). Only a small number of cells in this group had intact plasma membranes with smooth surfaces that were only covered with single short microvilli (data not shown). In samples cultivated for 2 and 24 h prior to cryopreservation, the same thin long fibers present in non-frozen control scaffolds were seen (Figs. 5d, 6d). This effect was not detected in samples after 0.5 h cultivation (Fig. 4d).

In general after 24 h recovery, hMSCs were covered with microvilli and some blebs and vesicles, and appeared mostly star-shaped (see Figs. 4e, 5e, 6e). However, in scaffolds with 24 h pre-cultivation the cells with disrupted plasma membranes were still seen after freezing-thawing procedure (not shown). Also the above-mentioned thin fibers were present in all cultivation groups (Figs. 4f, 5f, 6f).

Finally after 48 h cell recovery, most cells in every cultivation group possessed elongated star-shaped forms and were mostly covered with microvilli and some single vesicles (Figs. 4g, 5g, 6g). However, cells cultivated 0.5 h prior to cryopreservation appeared slimmer than in other groups, and cell–cell contacts were not dense (Fig. 4g). In contrast, discrimination of single cells was more difficult in the 2 h and 24 h cultivation groups due to tight cell–cell contacts (Figs. 5g, 6g). Remarkably, in all samples after 48 h recovery, fine filaments on the substrate were present (Figs. 4h, 5h, 6h).

### Effect of cryopreservation on the cell spreading

**Fig. 7:**
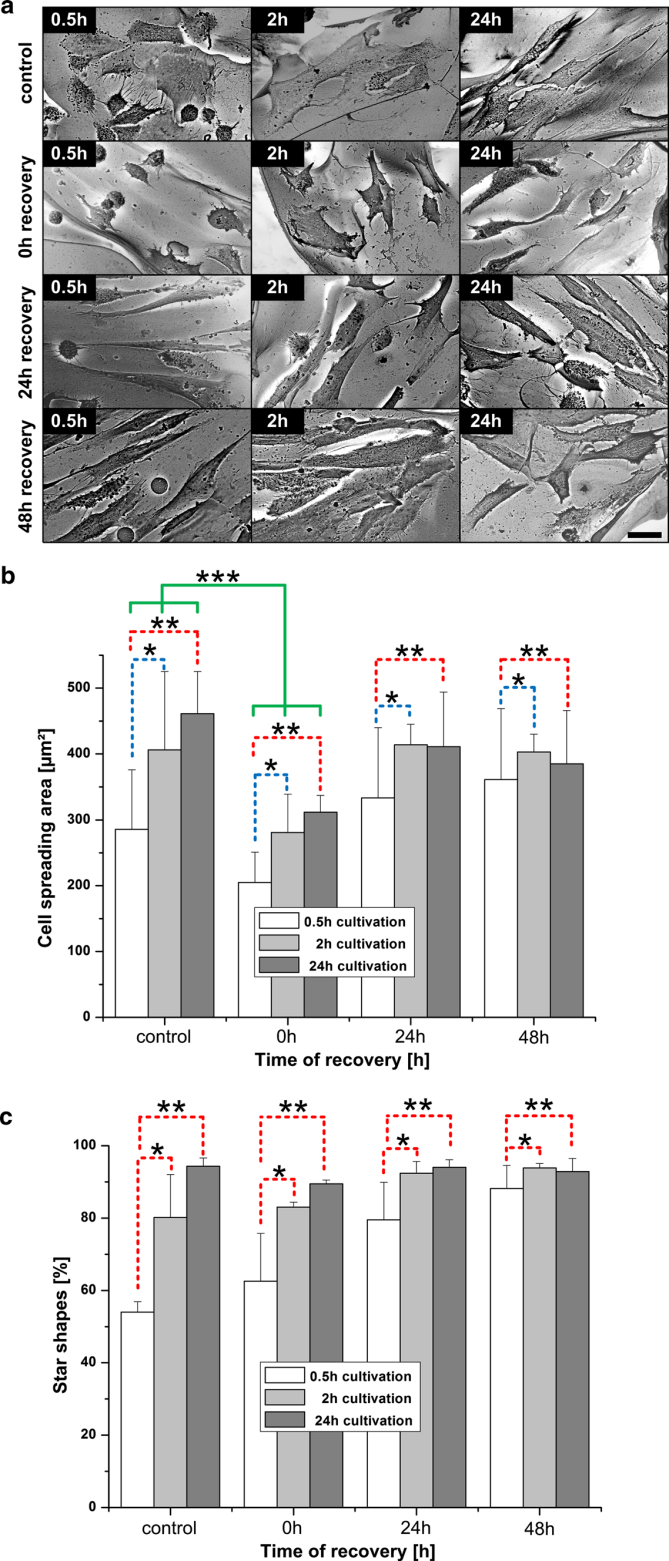
Cell spreading and cell shape determination (*n* = 3): **a** Representative, inverted BSE-images for calculation of hMSCs spreading area; *scale bar* 20 μm. **b** Spreading area before and after cryopreservation of hMSCs on alginate sponges. The differences between 0.5 and 2 h cultivation (*), as well as 0.5 versus 24 h cultivation (**) were statistically significant at every recovery time. Also, differences between control and 0 h recovery (***) were statistically significant at every cultivation time. *, **, and *** show *P* < 0.05. **c** Percentage of cells with *star shapes*. The differences between 0.5 versus 2 h (*), as well as 0.5 versus 24 h cultivation (**) were statistically significant at every recovery time (*P* < 0.05)

For investigation of cell spreading and calculation of cell area we used BSE mode of scanning electron microscope. The applied invert BSE-imaging method and its corresponding preparation enhanced contrast between cells and surfaces (Fig. 7a) and improved automatic or manual evaluation. Immediately after thawing we observed a significant decrease of the cell spreading area in all groups (Fig. 7a, b, 0 h recovery). Also in BSE-images, the cells cultivated for 0.5 h prior cryopreservation appeared slimmer than in all others groups. hMSCs cultivated for 2 h and 24 h prior to cryopreservation were mainly star-shaped with a minor number of round cells. The cell area of 0.5 h cultivation group was significantly less than at all recovery time points after cryopreservation (Fig. 7b). The cells cultivated for 2 h before cryopreservation showed a cell spreading area comparable to the non-frozen control scaffolds after 24 h recovery. From the same SEM-images we have calculated the percentage of star-shaped cells (Fig. 7c). The number of star-shaped cells in the 0.5 h cultivation group has always been significantly lesser than in all other groups. Only cells cultivated for 2 h and 24 h prior to cryopreservation showed cell shapes comparable to the non-frozen control after a recovery times of 24 and 48 h after thawing.

## Discussion

For the first time, we showed successful cryopreservation of adherent hMSCs within alginate-gelatin scaffolds with the potential to serve as a ready-to-use tissue engineered product. We observed that cryopreservation success depends on the cultivation time before cryopreservation and identified an optimal time period in order to recover adherent cells with maintained functionality.

### Short cultivation time is preferable for viability and recovery

In comparison with the work of Xu et al. [35], viability of cryopreserved hMSCs adherent to scaffolds could be enhanced after short time cultivation. Improved results of this work maybe due to the following reasons. First, the alginate-gelatin scaffolds used here are soft. Mechanical strains between shrinking cells and stiff scaffolds during cooling and ice crystallization occur in lesser amount, as both have similar thermal contractions, so that cells do not detach [35, 50]. Second, alginate as a potent hydrogel absorbs water and thus can avoid harmful formation of large ice crystals. The scaffolds remain soft and elastic after cryopreservation, and SEM images in Figs. 4, 5 and 6 do not show visible structural changes (ruptures or damages) between cryopreserved scaffolds and non-frozen controls.

Most research in cryopreservation of adherent cells use cultivation times of 24 h up to 7 days prior to freezing [32, 35, 40, 51–53]. However, our results show that the longest cultivation time (24 h) yielded lowest viability of hMSCs after thawing (Fig. 3b). Cell viability and the relative number of recovered cells with shorter cultivation time were significantly higher.

### Cytoskeleton, cell-substrate contacts and plasma membrane behavior influence on cryopreservation success

#### Major cell membrane/cytoskeleton disruptions after 24 h cultivation

After 24 h cultivation hMSCs were more spread than the cells in other groups and formed closed cell–cell and cell-substrate contacts mediated by integrins [30] and cytoskeletal structures such as actin stress fibers [53, 54]. Former studies already showed that rigidity of plasma membrane and cytoskeleton play key roles in freezing and thawing of adherent cells [35, 40]. Disruption of the cytoskeleton by ice crystal formation during cryopreservation can lead to cell detachment with subsequent cell death [35, 39, 40, 50]. Our results of hMSCs cultivated for 24 h before cryopreservation confirmed this thesis (see cell number, Table 1). We did not evaluate cytoskeletal proteins as did others [35, 50], but investigated cell state after cryopreservation by high-resolution SEM and checked cell spreading, shape and membrane integrity (ruptured and wrinkled plasma membranes, Figs. 4c, d, 6c). That allows an indirect proof of cytoskeleton breakdown of cells with maintained cell-substrate focal contacts.

#### Major cell loss after 0.5 h cultivation

After a short cultivation (0.5 h) of hMSCs, the number of viable attached hMSCs was higher than in scaffolds with 24 h cultivation time immediately after thawing, but was drastically reduced for increasing recovery times (compare number of living cells at each time of recovery with total cell number in control, Table 1). Most likely, the round-shaped, non-spread cells, predominated in scaffolds with 0.5 h cultivation time, had less focal contacts and therefore weak adhesion.

#### Shrinkage and cell-volume regulation

Another indirect measurement of cytoskeleton was performed by calculating the spreading area of cells. We observed a significant decrease of the spreading area in every group of cultivation immediately after thawing, what indicated the reduced cell volume after cryopreservation. During slow-freezing of cells, osmotic shrinkage occurs [55] what can lead to cell damage with following post-thaw death, if the cell is dehydrated over its hypothesized minimum [56]. The importance of cytoskeleton in cell volume regulation is well-known [57] and is discussed in cryopreservation with quick responses of membrane-cytoskeleton complex systems [58, 59]. Due to the water loss during cryopreservation, cell volume is reduced but there is membrane material still available (i.e. the cell membrane is developed and stiff), the cell can be recovered or repaired [59].

After 0.5 h and partially after 2 h cultivation, we observed round and flower-shaped hMSCs on non-frozen samples, covered with many microvilli, ruffles and small vesicles (see Fig. 4b). It is possible, that the developed membrane structures with enhanced mechanical properties [59] lead to higher stability of these cells to extracellular ice damage and therefore increased number of viable cells immediately after thawing (see Fig. 3b). Another possible explanation for the enhanced viability of cells cultivated for 0.5 and 2 h prior to cryopreservation in comparison to 24 h cultivation group is the cytoskeleton stiffness: at a reduced cell volume and increased cytoskeleton stiffness of cells cultivated 24 h before freezing irreversible damage and cell death followed [59].

We measured a major reduction of cell area directly after thawing and removal of the hyperosmotic cryoprotectant DMSO (see Fig. 7b). DMSO removal processes take approximately 10 min, but the cellular adaptation with regulatory processes after hyperosmotic stress takes 0–20 h (for actin 10–30 min) [60]. However, in a recent published paper, real-time osmotic response of adherent cells was measured with approximately only 5 min [61]. Most likely, we measured cells immediately at 0 h recovery, so that they did not finish their volume regulation procedure.

#### Influence of cultivation time before cryopreservation on cell-shape/differentiation

As some publications already showed, the shape of hMSCs reflects their future differentiation possibilities [49, 54]. According to this we can speculate that hMSCs with round morphology after 0.5 h cultivation before cryopreservation (i.e. reduced number of star-shaped cells, see Fig. 7c) could be more preferable to adipogenic and chondrogenic pathways of differentiation. After 2 and 24 h cultivation, more cells show star-shaped morphology, which might be beneficial for differentiation of hMSCs in osteoblasts.

### Maintenance of functionality by migration of hMSCs on scaffolds after cryopreservation

Since the ability of hMSCs to migrate is of great importance in tissue repair and regeneration [62, 63], it is crucial to maintain this potential for future stem cell transplantation [64]. Therefore we evaluated the cell motility in SEM images by so-called cell traces closely connect with cytoskeleton proteins (tubulin, actin etc.) as well as focal contact proteins (i.e. integrins) [63, 65, 66]. Such traces were visible in our samples: we observed fine, long fibers throughout the scaffolds that, according to morphology and size, can be classified as cell traces. These traces were observed when hMSCs were cultivated for 2 or 24 h before cryopreservation (Figs. 5b–h, 6b–h). After a short cultivation (0.5 h) such traces could not be observed in non-frozen scaffolds and immediately after thawing. In general, the availability of cell traces may be a significant characteristic of cell migration indicating the physiological and functional activity of cells within scaffolds [62, 63]. These results serve as basis for future experiments and applications, especially for wound healing.

### Discussion of analyzing procedures

#### Standard deviation

High standard deviations in our cell area measurements occur due to following reason. We have observed different processes: some cells were newly attached and therefore still spherical (0.5 h and, partially, 2 h), others already begun to spread (0.5 and 2 h cultivation), were already spread (24 h), or had already divided (especially 24 and 48 h recovery in all groups), resulting in a decreased cell spreading area [67].

#### Cell number in control

Considering cell spreading area, morphology and cell traces of adherent hMSCs within alginate-gelatin scaffolds, cells cultivated for 2 h before cryopreservation showed fewest deviation and best viability relative to the non-frozen control scaffolds. However, total cell number after 2 h cultivation in non-frozen controls was less than after other cultivation times. Possible reasons for that might be following: as can be seen in Figs. 3a and 4, and in consistency with former work of Anselme et al. [42, 68], cells after 0.5 h cultivation time were initially adhesive and only minor spread. The viability assay for those scaffolds was performed immediately and before addition of culture medium. The addition of medium to groups of 2 and 24 h cultivation to prevent drying rinsed the minor spread cells from the alginate-gelatin scaffold. Most likely, this cell detachment causes the low total cell number after 2 h of cultivation, but the cells cultivated for 24 h had enough time to divide and double. To eliminate this artifact, we normalized the cell number to the initial seeded cells. This phenomenon needs further investigation, since an improved cultivation time between 2 and 24 h seems possible.

### Significance of ready to-use cryogel scaffolds for regenerative medicine

It has been reported that cryogels have significant characteristics for cell-based biomedical applications [23, 27, 28, 69]. First to mention is the good adhesion potential of the sponges resulting in cell growth, proliferation and migration [31]. With electron scanning microscopy we observed cell traces and showed here, that the chemical coupling of gelatin to alginate [19] enables an excellent adhesion of therapeutically interesting mesenchymal stem cells. Secondly, cryogels with pore sizes up to 100 μm allow cell–cell connections and cell-environment interactions like proliferation, migration and signaling [28, 31, 69, 70], which we could proof by laser scanning microscopy. These properties may lead the way for applications in wound healing and tissue repair. The third important fact is their elasticity and high water absorbing capacity [12, 70, 71], what can avoid cell and substrate damage caused by ice crystallization during cryopreservation processes. Altogether we showed here, that the elastic properties might benefit cryopreservation procedures, that adhesion potential is still intact after cryopreservation procedures and that large pore size allows good cell migration and diffusion of (cryo-)media. Finally, the shown determination of cell shape (round or elongated) can be modulated and screened by cultivation time and/or adjustable stiffness of the cryogels, leading the way to ready-to-use scaffolds for regenerative medicine and capable of long-time storage.

## Conclusions

We compared the influence of different cultivation times (0.5, 2, 24 h) before cryopreservation of adherent hMSCs within alginate-gelatin scaffolds on viability, morphology and recovery at different time points (0, 24 and 48 h) after thawing. Criteria of cryopreservation success were: (a) cell viability; (b) number of adherent, viable cells; (c) cell morphology and form; (d) cell spreading area; (e) the ability of cells to migrate. For the first time, we showed that short cultivation times (0.5 and 2 h) are more preferable for adherent hMSCs cryopreservation than a long cultivation time (24 h): a short cultivation time yielded higher viability and recovery what was confirmed by the cell’s ability to expand and migrate. More precisely, hMSCs cultivated for 2 h before cryopreservation recovered most rapidly from freezing and showed a higher percentage of viable cells than cells cultivated for only 0.5 h.

Our work proofs that adherent hMSCs on alginate-gelatin scaffolds can be cryopreserved successfully without any further preparation steps and thus build a valuable tool for future regenerative medicine. This is a first step towards ready-to-use 3-D scaffolds for long-time storage at cryogenic temperatures in biobanks.
